# Supplementary material for: Gibberellins promote polar auxin transport to regulate stem cell fate decisions in cambium
Source: Nat Plants. 2023 Mar 30;9(4):631–44. doi: 10.1038/s41477-023-01360-w (PMC10119023; doi:10.1038/s41477-023-01360-w)
Supplement: Supplementary file 1 — Supplementary Tables 1–4. [file 41477_2023_1360_MOESM1_ESM.pdf]

nature plants

Article

https://doi.org/10.1038/s41477-023-01360-w

Gibberellins promote polar auxin transport to regulate stem cell fate decisions in cambium

In the format provided by the authors and unedited

Table S1. Primers used in cloning

primer name	Sequence (5'-3')	to clone construct
RGAd17 FP attb1	GGGGACAAGTTTGTACAAAAAAGCAGGCTCGATGAAGAGAGATCATCAC	221z-RGAd17
RGAd17 RP attb2	GGGGACCACTTTGTACAAGAAAGCTGGGTCGTACGCCGCCGTCGAGAGTTTCCA	221z-RGAd17
BSAI-DR5V2-F1	TTCAGAggtctcTctcgGAATTCCCGACAAAAG	1R4z-DR5v2
BSAI-DR5V2-R1	AGCGTGggtctcGttgcGGATCCCCTGTAATTGTAATTG	1R4z-DR5v2
**genotyping primers**		
pin1-1 FP	ATGATTACGGCGGCGGACTTCTA	
pin1-1 RP	TTCCGACCACCACCAGAAGCC	
pin1-7 SALK FP	CAAAAACACCCCCAAAATTTC	
pin1-7 SALK RP	AATCATCACAGCCACTGATCC	
ga1_LP	CAGACCCGAGACAGTAACTGC	
ga1_RP	TCTCTACTCGAGGCAAGCTTG	
SALK_LBb1.3	ATTTTGCCGATTTCGGAAC	
**qRT-PCR primers**		reference
ACT2	ACATTGTGCTCAGTGGTGGA<br>CTGAGGGAAGCAAGAATGGA	Smetana et al. 2019
TIP41	GTGAAAACTGTTGGAGAGAAGCAA<br>TCAACTGGATACCCTTTCGCA	Han et al. 2013
UBQ10	CGTCTTCGTGGTGGTTTCTAAA<br>TACAAGGCCCCAAAACACAAA	Smetana et al. 2019
ATHB8	AACACCACTTGACCCCTCAACATCAG<br>CACGCAACCAACAAGGCTTATCC	Carlsbecker et al. 2010
ABCB21	TCGCTCATACGTCTACAAGAAGATACTAAACA<br>CGAAAGAGACTTTCTTTTCTTTGATCGG	Kamimoto et al. 2012

Table S2. Entry clones used in this study

Entry clones	origin	Note
221z-RGAd17	this study	template plasmid (RGAd17-GR) for PCR received from Laura Ragni
2R3e-4xgly-TagRFP-OscT	Siligato et al. 2015	
pRU24_43GW	Wang et al. 2020	
1R4a-pPEAR1-XVE	Roszak et al. 2021	
1R4a-pANT-XVE	Siligato et al. 2015	
1R4a-pAtHB8-XVE	Smetana et al. 2019	
221z-CRE1	Smetana et al. 2019	
2R3e-nosT	Siligato et al. 2015	
pCAM-hyg-R4r3	Siligato et al. 2015	
1R4a-pAPL	Roszak et al. 2021	
221z-erRFP	Siligato et al. 2015	
2R3e-3AT	Siligato et al. 2015	
pBm43GW	Karimi et al. 2007	
1R4a-pANT	this study	
pCam-kan-R4R3	Siligato et al. 2015	
221z-erGUSpRFP	this study	
1R4z-DR5v2	this study	
2R3z-nosT	Siligato et al. 2015	
pHm43GW	Karimi et al. 2007	

Table S3. Expression vectors generated in this study

Full name	Name in the manuscript	1st box name	2nd box name	3rd box name	dest name
pRU24_43GW--pPEAR1-XVE-RGAd17-4xgly-TagRFP	*pPEAR1:XVE>>RGAd17*	1R4-pEPM-XVE	221z-RGAd17	2R3e-4xgly-TagRFP	pRU24_43GW
pRU24:43GW--pANT-XVE-RGAd17-4xgly-TagRFP	*pANT:XVE>>RGAd17*	1R4-pANT-XVE	221z-RGAd17	2R3e-4xgly-TagRFP	pRU24_43GW
pRU24_43GW--pAtHB8-XVE-RGAd17-4xgly-TagRFP	*pAtHB8:XVE>>RGAd17*	1R4a-pAtHB8-XVE	221z-RGAd17	2R3e-4xgly-TagRFP	pRU24_43GW
pCAM-hyg--pPEAR1:XVE>>CRE-nosT		1R4a-pPEAR1:XVE	221z-CRE1	2R3e-nosT	pCAM-hyg-R4R3
pBm43GW--pAPL:erRFP-3AT	*pAPL:erRFP*	1R4a-pAPL	221z-erRFP	2R3e-3AT	pBm43GW
pCAM-kan-R4R3--pANT:erGUSpRFP-3AT	*pANT:erGUSpRFP*	1R4a-pANT	221z-erGUSpRFP	2R3e-3AT	pCam-kan-R4R3
pHm43GW--DR5v2:erRFP-nosT	*DR5v2:erRFP*	1R4z-DR5v2	221z-erRFP	2R3z-nosT	pHm43GW

Table S4. Plant material used in this study

Seeds	Name in the manuscript	source	DOI
pHS:Dbox-CRE x 35S:lox-GUS	*HSdCR*	Smetana et al. 2019	DOI: 10.1038/s41586-018-0837-0
pHS:Dbox-CRE x 35S:lox-GUS x ga1	*HSdCE x ga1*	this study	
p35S:XVE>>miR165a	*p35S:XVE>>miR165a*	Smetana et al. 2019	DOI: 10.1038/s41586-018-0837-0
pPEAR1:XVE-CRE x 35S:lox-GUS	*pPEAR1:XVE>>CRE*	this study	
pANT:XVE-CRE x 35S:lox-GUS	*pANT:XVE>>CRE*	Smetana et al. 2019	DOI: 10.1038/s41586-018-0837-0
pPIN1:PIN1-GFP	*pPIN1:PIN1-GFP*	Xu et al. 2006	DOI: 10.1126/science.1121790
DR5v2:erRFP	*DR5v2:erRFP*	this study	
DR5rev:GUS	*DR5rev:GUS*	Baesso et al. 2018	DOI: 10.1111/plb.12711
pPIN1:PIN1-GFP x ga1	*pPIN1:PIN1-GFP x ga1*	Willige et al. 2011	DOI: 10.1105/tpc.111.086355
pPIN1:PIN1-GFP x arf7-1,19-1	*pPIN1:PIN1-GFP x arf7-1,19-1*	this study	
pPIN1:PIN1-GFP x DR5v2:erRFP	*pPIN1:PIN1-GFP x DR5v2:erRFP*	this study	
pPIN1:PIN1-GFP x DR5v2:erRFP x ga1	*pPIN1:PIN1-GFP x DR5v2:erRFP x ga1*	this study	
pPIN2:PIN2-GFP	*pPIN2:PIN2-GFP*	Xu et al. 2006	DOI: 10.1126/science.1121790
pPIN3:PIN3-GFP	*pPIN3:PIN3-GFP*	Zadnikova et al. 2010	DOI: 10.1105/tpc.106.048777
pPIN4:PIN4-GFP	*pPIN4:PIN4-GFP*	Blilou et al. 2005	DOI: 10.1038/nature03184
pPIN7:PIN7-GFP	*pPIN7:PIN7-GFP*	Blilou et al. 2005	DOI: 10.1038/nature03184
pAPL:erRFP	*pAPL:erRFP*	this study	
pANT:erGUSpRFP	*pANT:erRFP*	this study	
pANT:XVE>>RGAd17-TagRFP	*pANT:XVE>>RGAd17*	this study	
pAtHB8:XVE>>RGAd17-TagRFP	*pAtHB8:XVE>>RGAd17*	this study	
pPEAR1:XVE>>RGAd17-TagRFP	*pPEAR1:XVE>>RGAd17*	this study	
pRGA:GFP-RGA	*pRGA:GFP-RGA*	Silverstone et al. 2001	DOI: 10.1105/tpc.010047
rga-24,gai-16	*rga,gai*		

arf7-1,19-1	*arf7-1,19-1*	Okushima et al. 2005	DOI: 10.1105/tpc.104.028316
arf7-2,19-5	*arf7-2,19-5*	Goh et al. 2012	DOI: 10.1242/dev.071928
pUBQ10:XVE>>amiMP,arf7-2,19-5	*amiMP,arf7-2,19-5*	this study	
pin1-7 (SALK-047613)	*pin1-7*	Alonso et al. 2003	DOI: 10.1126/science.1086391
pin3,4,7	*pin3,4,7*	Govindaraju et al. 2020	DOI: 10.1242/dev.187666
pin1-1	*pin1-1*	Gälweiler et al. 1998	DOI: 10.1126/science.282.5397.2226
ga1 (SALK-109115)	*ga1*	Willige et al. 2011	DOI: 10.1105/tpc.111.086355
abcb19-101	*abcb19*	Lin & Wang 2005	DOI: 10.1104/pp.105.061572
abcb21-1	*abcb21*	Jenness et al. 2019	DOI: 10.3389/fpls.2019.00806.
