# Supplementary material for: Gibberellins promote polar auxin transport to regulate stem cell fate decisions in cambium
Source: Nat Plants. 2023 Mar 30;9(4):631–44. doi: 10.1038/s41477-023-01360-w (PMC10119023; doi:10.1038/s41477-023-01360-w)
Supplement: Supplementary file 2 — Reporting Summary [file 41477_2023_1360_MOESM2_ESM.pdf]

nature portfolio

Corresponding author(s): Ari Pekka Mähönen

Last updated by author(s): Jan 15, 2023

Reporting Summary

Nature Portfolio wishes to improve the reproducibility of the work that we publish. This form provides structure for consistency and transparency in reporting. For further information on Nature Portfolio policies, see our Editorial Policies and the Editorial Policy Checklist.

Statistics

For all statistical analyses, confirm that the following items are present in the figure legend, table legend, main text, or Methods section.

n/a	Confirmed	
☐	☒	The exact sample size (*n*) for each experimental group/condition, given as a discrete number and unit of measurement
☐	☒	A statement on whether measurements were taken from distinct samples or whether the same sample was measured repeatedly
☐	☒	The statistical test(s) used AND whether they are one- or two-sided<br>*Only common tests should be described solely by name; describe more complex techniques in the Methods section.*
☒	☐	A description of all covariates tested
☐	☒	A description of any assumptions or corrections, such as tests of normality and adjustment for multiple comparisons
☐	☒	A full description of the statistical parameters including central tendency (e.g. means) or other basic estimates (e.g. regression coefficient) AND variation (e.g. standard deviation) or associated estimates of uncertainty (e.g. confidence intervals)
☐	☒	For null hypothesis testing, the test statistic (e.g. *F*, *t*, *r*) with confidence intervals, effect sizes, degrees of freedom and *P* value noted<br>*Give P values as exact values whenever suitable.*
☒	☐	For Bayesian analysis, information on the choice of priors and Markov chain Monte Carlo settings
☒	☐	For hierarchical and complex designs, identification of the appropriate level for tests and full reporting of outcomes
☒	☐	Estimates of effect sizes (e.g. Cohen's *d*, Pearson's *r*), indicating how they were calculated

Our web collection on statistics for biologists contains articles on many of the points above.

Software and code

Policy information about availability of computer code

Data collection	Leica LAS X, Leica LAS x lite, Fiji 1.53, Bio-Rad CFX Manager
Data analysis	R version 4.1.2, R-studio 2022.07.2 , Python 3.8.10, Fiji 1.53, Leica LAS X, MS Excel Professional Plus 2016, Adobe Photoshop, https://github.com/LMIVainio/PolarUnwrap/find/main ,

For manuscripts utilizing custom algorithms or software that are central to the research but not yet described in published literature, software must be made available to editors and reviewers. We strongly encourage code deposition in a community repository (e.g. GitHub). See the Nature Portfolio guidelines for submitting code & software for further information.

Data

Policy information about availability of data

All manuscripts must include a data availability statement. This statement should provide the following information, where applicable:

- Accession codes, unique identifiers, or web links for publicly available datasets
- A description of any restrictions on data availability
- For clinical datasets or third party data, please ensure that the statement adheres to our policy

All data supporting the findings of this article are available in this article and its supplementary information. Source data are provided with this paper. All lines involved in this study are available upon reasonable request from the corresponding author.
Gene Accession numbers for Genes used in this study is: CYCB1;1, AT4G37490; PEAR1, AT2G37590; ANT, AT4G37750; AtHB8, AT4G32880; MIR165A, AT1G01183;

MP, AT1G19850; ARF7, AT5G20730; ARF19, AT1G19220; GA1, AT4G02780 ; RGA, AT2G01570; PIN1, AT1G73590; GAI, AT1G14920; PIN2, AT5G57090; PIN3, AT1G70940; PIN4, AT2G01420; PIN7, AT1G23080; APL, AT1G79430; ABCB19, AT3G28860; ABCB21, AT3G62150.
Data for Extended data Fig 7a comes from eFP browser "http://bar.utoronto.ca/efp2/Arabidopsis/Arabidopsis_eFPBrowser2.html" (ref 76) and in Goda et. al. 2008 " (ref 77) https://doi.org/10.1111/j.1365-313X.2008.03510.x"

Human research participants

Policy information about studies involving human research participants and Sex and Gender in Research.

Reporting on sex and gender	n.a.
Population characteristics	n.a.
Recruitment	n.a.
Ethics oversight	n.a.

Note that full information on the approval of the study protocol must also be provided in the manuscript.

Field-specific reporting

Please select the one below that is the best fit for your research. If you are not sure, read the appropriate sections before making your selection.

☒ Life sciences ☐ Behavioural & social sciences ☐ Ecological, evolutionary & environmental sciences

For a reference copy of the document with all sections, see nature.com/documents/nr-reporting-summary-flat.pdf

Life sciences study design

All studies must disclose on these points even when the disclosure is negative.

Sample size	No statistic method was used to predetermine the sample size. For all the experiments, we first performed a preliminary analysis with a few samples, and this preliminary analysis was subsequently confirmed with a larger sample size, which was comparable with previous studies (e.g Miyashima et al. 2019 Nature).
Data exclusions	We excluded samples that germinated poorly, or showed overall growth defects that were confirmed genetically not to be related to the genotype. These defects occur occasionally, and are caused by the seed sterilization method used.
Replication	All experiments were repeated at least two times and one representative repeat was shown in the figures.
Randomization	No randomization was applied, but seedlings of different genotypes were grown together in same growth chambers.
Blinding	We did not use blinding. In order to get as objective results as possible, in multiple experiments we had other researchers repeating the experiments.

Reporting for specific materials, systems and methods

We require information from authors about some types of materials, experimental systems and methods used in many studies. Here, indicate whether each material, system or method listed is relevant to your study. If you are not sure if a list item applies to your research, read the appropriate section before selecting a response.

Materials & experimental systems

n/a	Involved in the study	
☒	☐	Antibodies
☒	☐	Eukaryotic cell lines
☒	☐	Palaeontology and archaeology
☒	☐	Animals and other organisms
☒	☐	Clinical data
☒	☐	Dual use research of concern

Methods

n/a	Involved in the study	
☒	☐	ChIP-seq
☒	☐	Flow cytometry
☒	☐	MRI-based neuroimaging
